# Expression and functional implications of the renal apelinergic system in rodents

**DOI:** 10.1371/journal.pone.0183094

**Published:** 2017-08-17

**Authors:** Anne-Marie O’Carroll, Sabrine Salih, Philip R. Griffiths, Aarifah Bijabhai, Mark A. Knepper, Stephen J. Lolait

**Affiliations:** 1 Bristol Medical School, HW-LINE, University of Bristol, Bristol, United Kingdom; 2 Epithelial Systems Biology Laboratory, Systems Biology Center, National Heart, Lung and Blood Institute, National Institutes of Health, Bethesda, Maryland, United States of America; George Washington University School of Medicine and Health Sciences, UNITED STATES

## Abstract

Apelin binds to the G protein-coupled apelin receptor (APJ; gene name *aplnr*) to modulate diverse physiological systems including cardiovascular function, and hydromineral and metabolic balance. Recently a second endogenous ligand for APJ, named apela, has been discovered. We confirm that apela activates signal transduction pathways (ERK activation) in cells expressing the cloned rat APJ. Previous studies suggest that exogenous apela is diuretic, attributable wholly or in part to an action on renal APJ. Thus far the cellular distribution of *apela* in the kidney has not been reported. We have utilized *in situ* hybridization histochemistry to reveal strong *apela* labelling in the inner medulla (IM), with lower levels observed in the inner stripe of the outer medulla (ISOM), of rat and mouse kidneys. This contrasts with renal *aplnr* expression where the converse is apparent, with intense labelling in the ISOM (consistent with vasa recta labelling) and low-moderate hybridization in the IM, in addition to labelling of glomeruli. *Apelin* is found in sparsely distributed cells amongst more prevalent *aplnr*-labelled cells in extra-tubular regions of the medulla. This expression profile is supported by RNA-Seq data that shows that *apela*, but not *apelin* or *aplnr*, is highly expressed in microdissected rat kidney tubules. If endogenous tubular apela promotes diuresis in the kidney it could conceivably do this by interacting with APJ in vasculature, or via an unknown receptor in the tubules. The comparative distribution of *apela*, *apelin* and *aplnr* in the rodent kidney lays the foundation for future work on how the renal apelinergic system interacts.

## Introduction

The G protein-coupled apelin receptor (APJ; gene name *aplnr*) is activated by the endogenous ligand apelin [1,2]. The apelinergic system has been implicated in a wide range of homeostatic processes, including cardiovascular control/cardio-embryogenesis, angiogenesis, hydromineral balance, hypothalamic-pituitary-adrenal axis regulation, and metabolic homeostasis that may involve a pathophysiological role in obesity [2].

As a hormone in the periphery or neuromodulator in the brain, *apelin* appears to be co-localized with APJ in some brain regions such as the paraventricular and supraoptic nuclei of the hypothalamus, and in many peripheral tissues including anterior pituitary, lung, heart, kidney, stomach and mammary glands, where it may act in an autocrine or paracrine fashion [2]. The tissue expression of *aplnr* is largely supported by the receptor autoradiographical localization of APJ-binding sites [3–5].

Apelin exists as a number of proteoforms (i.e., [Pyr^1^]apelin-13, apelin-17, apelin-36) that derive from post-translational modification of a 77 amino acid apelin pro-hormone precursor. Upon binding APJ, apelin activates a myriad of signal transduction pathways that are often G protein-dependent, including inhibition of adenylate cyclase, stimulation of MAP kinase (ERK) phosphorylation, Akt phosphorylation that could contribute to neuroprotection, and enhancing nitric oxide synthase activity [2]. APJ activation by apelin-13 may involve multiple G proteins [6], while some actions involving APJ may be apelin-independent (e.g., G protein-independent myocardial cell hypertrophy response to mechanical stretch) or apelin-dependent (e.g., blunting myocardial hypertrophy) in the same tissue [7].

A second endogenous ligand for APJ has recently been discovered [8,9]. Originally annotated as a long non-coding (nc) RNA (termed *Ende* [10]) expressed primarily in the definitive endoderm during mouse embryogenesis, the *apela* (otherwise known as *elabela* or *toddler*) gene is also translated [8,9]. It encodes a predicted 54 (58 in zebrafish) amino acid precursor molecule that is highly conserved across vertebrates and is structurally distinct from the apelin prohormone [8,9]. The apela precursor can be enzymatically processed into a number of biologically active proteoforms such as apela-21 and apela-32 that may be secreted [8,9]. Apela-32 binds to APJ with an affinity (nanomolar) similar to apelin and may be more potent than apelin in activating APJ signal transduction pathways in some APJ-expressing cells [11,12]. Apela is essential for cell movement during gastrulation and in the development of zebrafish heart and vasculature where loss of apela function usually results in embryonic lethality that can be rescued by injection of *apela* mRNA [9]. Zebrafish *apela* null mutants invariably exhibit cardiac dysplasia [8,9,13], reminiscent of the phenotype observed in the majority of embryonic *aplnr* knockout mice [14]. Since cardiac abnormalities are not observed in embryonic *apelin* knockout mice [14,15], it is possible that apela may be the main endogenous APJ ligand during mouse cardiovascular development. Interestingly heart defects (or complete absence of cardiomyocytes) are also observed when *apela* or *apelin* are over-expressed in zebrafish [9,16], indicating that normal levels of either APJ ligand are obligatory for proper heart development.

Exogenously administered apela is anorexigenic [17], and promotes diuresis, presumably by direct activation of APJ located in the kidney and/or indirectly by regulating arginine vasopressin expression [11] in the hypothalamus. Apela activates the PI3K/Akt pathway and is anti-apoptotic in human embryonic stem cells (ESCs) even though these cells did not express *aplnr* [18], while apela-induced p53-mediated apoptosis in mouse ESCs points to a RNA-regulatory rather than a protein-coding function [19]. This depends on *apela* binding heterogeneous nuclear ribonucleoprotein L (hnRNPL), an inhibitory binding partner of p53 in these cells. Whether apela acts as a protein in a non-APJ-dependent manner (e.g., via an unrelated receptor) or as a regulatory RNA *in vivo* is not known.

*Apela* mRNA transcripts appear to be more prevalent during development [8,9,10]. They are predominantly expressed in the heart of embryonic rodents [10] and zebrafish [8,9], and are also detected in isolated cells from the adult rat heart [20], and in adult rat and mouse kidney [10,11] by RT-PCR. The precise renal location of *apela* gene expression in the kidney has not been reported. Evidence for renal *apelin* expression is less convincing—it appears to be sparsely distributed in tissue endothelial cells and perhaps vascular epithelial cells and glomeruli [21]. In the rat and mouse kidney *aplnr* is mainly expressed in vascular elements (vasa recta) of the outer medulla and glomeruli [5,22,23]—lower levels have been described in the rat collecting ducts as determined by RT-PCR of isolated tubule segments [22]. It has been proposed that apelin-17, acting on the collecting duct APJ, counteracts the antidiuretic effect of arginine vasopressin acting at the V2 receptor [24].

The aims of this study are to confirm apela bioactivity via APJ *in vitro* and to characterize the anatomical relationship between *apela* and *aplnr* expression in the rat and mouse kidney using *in situ* hybridization histochemistry (ISHH) with gene-specific oligonucleotides. In an attempt to gain further insights into the possible functions of the renal apelinergic system we have also utilized branched-chain ISHH to determine whether the *apelin*, *apela* and/or *aplnr* genes are co-expressed in the same regions in the rat kidney. This distribution is supported by RNA-Seq studies on isolated renal tubule segments. Our studies reveal that while *apela* and *aplnr* expression overlap, *apela* is mainly expressed in the medullary collecting ducts and loops of Henle whereas *aplnr* is highly expressed in non-tubular structures in the outer medulla. *Apelin* expression is largely restricted to isolated cells mainly in the vicinity of *aplnr* cells in the medulla. This distribution raises questions about how the two-ligand apelinergic system operates in regulating renal function.

## Materials and methods

### Animals

Adult (8–12 weeks old) male wildtype (n = 3) or null mutants (n = 2) mice from our *aplnr* knockout colony [25] (mixture of C57BL/6 x 129X1/Sv strains) and adult (approx 275g) male Wistar rats (n = 8) were used in this study (Sprague-Dawley rats were used for RNA-Seq—see below). Animals were housed under constant temperature (21±2°C), light (lights on from 0700 to 1900h) and humidity (45–50%) regimens with food and water *ad libitum*. Animal care and maintenance were performed in accordance with the Animal Scientific Procedures Act (1986) United Kingdom and approved by the Bristol University Animal Welfare and Ethical Review body.

### Cells

Chinese hamster ovary (CHO) cells were transfected with the rat *aplnr* cDNA (B78 [23]) as previously described [26]. Stably-transfected clonal cells were obtained by limiting dilution, and expression of *aplnr* was determined by Northern dot blots using two ^32^P-dATP-labelled 48bp oligonucleotide specific for the rat *aplnr* sequence (B78A and B78B [23]; see ISHH below). The highest *aplnr*-expressing cell clones were expanded for use, and one line (CHO-B78) was used for branched-chain *in situ* hybridization histochemistry and ERK signal transduction assays (see below).

### ERK immunohistochemistry and analysis

CHO-B78 cells were grown in 96-well plates at a concentration of approx 10,000 cells/well and serum-starved (0.1% foetal calf serum) O/N. Dose-response curves were performed for 10min, where [Pyr^1^]apelin-13 stimulation of ERK1/2 is maximal as established in pilot studies on CHO-B78 cells. After incubation with 0.01-1000nM [Pyr^1^]apelin-13 (Bachem) or rat apela-32 (cyclized, pyroglutamated; Severn Biotech, U.K.), or vehicle (dH_2_O), the cells were immunostained for dual phosphorylated(pp) ERK1/2 and total(t) ERK1/2—cell images were acquired using the IN Cell Analyzer 1000 and analysis performed by In Cell Analyzer Workstation 3.5 software (GE Healthcare) as previously described [27,28]. The nuclear and cytoplasmic fluorescence intensities in individual cells were quantified (in arbitrary units)—data was normalized to vehicle controls after subtraction of ‘no primary antibody’ backgrounds. In each experiment the data (nuclear + cytoplasmic ppERK measurements) is mean ± SEM, n = 6 (approx 1200 cells imaged from 3 wells of a 96-well plate; experiments repeated twice).

### *In situ* hybridization histochemistry (ISHH)

#### ISHH with radiolabelled oligonucelotides

Kidneys were frozen on powdered dry-ice and stored desiccated at -80°C prior to processing. Sections (12μm) were thaw-mounted onto polylysine-coated slides and ISHH performed with two ^35^S-end-labelled 48bp-oligonucleotides targeting *apela*, as described in detail for oligonucleotide probes (http://www.wsyacy.com/SNGE/Protocols/ISHH/ISHH.html). The antisense probes used in this study were rAP-1 and rAP-2 specific for rat *apela* (bp495-542 and bp849-896, respectively, in the 3’-untranslated region of GenBank Accession XM_008772035 (LOC100912649)), and mAP-1 and mAP-2 specific for mouse *apela* (bp481-528 and bp832-879, respectively, in the 3’-untranslated region of GenBank Accession NR_040692). ISHH with rat *aplnr* probes (B78A and B78B directed to bp602-649 and bp886-933 of the rat *aplnr*, GenBank Accession NM_031349, respectively [23]) was used to compare the expression of *aplnr* with *apela* in rat kidney.

Corresponding sense probes used as negative controls gave little, or low, levels of uniform background labelling.

Sections were exposed to X-ray film (Hyperfilm MP, Amersham) for 1–3 months at RT or apposed to emulsion (Ilford K5) for up to a year at 4°C (by which time the signal was saturated). Film images were scanned and pseudocoloured in Image J by inverting LUX of ‘16 colours’ in Lookup Tables. Emulsion-coated sections were developed manually with D-19 developer (Agar Scientific Ltd. U.K.) according to the manufacturer’s instructions, counterstained with toluidine blue and viewed on a Leica DM2000 microscope equipped with a Lecia DFC70000T camera and Leica Application Suit X (LAS X) workstation.

#### Branched-chain ISHH

To compare the expression of *apela*, *apelin* and *aplnr* in the same rat heart or kidney sections RNAscope (Advanced Cell Diagnostics; ACD) with the Multiple Fluorescence Assay kit was used. In this technique, target-specific Z-shaped probes with overhangs create a ‘bridge’ that binds a preamplifier that can then bind multiple amplifiers. The technique provides single RNA copy detection [29], typically ‘dots’, and permits rapid visualization (1 day) of low levels of mRNA (as is often observed for G protein-coupled receptors (GPCRs)).

Three fluorescent colour combinations were available, Alexa 488 (green), Atto 550 (orange) and Atto 647 (Far red), the colour for each target probe set dependant on which fluorescent colour modules (Amp4 AltA-C) and channels (C1-3) are used. The proprietary probes were designed by ACD for the following rat sequences: *apela* (GenBank Accession XM_008772035 (LOC100912649); bp147-1053; 12 Z probe pairs), *apelin* (NM_031612; bp2-996; 20 Z probe pairs) and *aplnr* (NM_031349; bp147-1053; 20 Z probe pairs). RNAscope was performed on 16μm frozen sections that were post-fixed in 4% PFA, according to the ACD user protocols (sheets#320513 and 320293). RNAscope detection of *aplnr* expression in CHO-APJ (B78) and nontransfected CHO cells (grown at a density of ~125,000–175,000 cells/12-well plate containing a glass 13mm coverslip (type 1) for 2 days) followed the tissue section protocol with the exception that pre-treatment was performed as in ACD sheet#320528. With cells, only the aplnr probe(s) (and positive and negative probes in parallel) were used, with a blank probe in the other two channels.

Positive (rat *POLR2A*, *PPIB* and ubiquitin (*UBC*); 3-plex probe set; ACD#407301) and negative (*DapB*; ACD#320871)) probes obtained from ACD were included in all experiments. Sections (and cells) were counterstained with DAPI as per ACD instructions.

The fluorescence signal in heart ventricles and throughout the kidney was visualized under a x40 objective on a Leica SPE single channel confocal laser scanning microscope attached to a Leica DMi8 inverted epifluorescence microscope equipped with a Lecia DFC365FX monochrome digital camera and LAS X workstation (University of Bristol Wolfson Bioimaging Centre). Z stacks were taken of all images with a Z step size of 0.5 μm. Images were exported to Image J (where Far-red labelling was sometimes converted to white) or Adobe Photoshop. No attempt was made to quantitate the amount of ‘dot’ labelling.

### Mining of transcriptome (RNA-Seq) data of microdissected rat renal tubules and glomeruli

Previously unpublished high-throughput, ‘deep’ sequencing (RNA-Seq) data was retrieved from a dataset on microdissected rat kidney tubules from adult male Sprague-Dawley rats [30]. The precision of the technique and accuracy of the dissection was verified by establishing the gene expression levels of several water and solute transporters with known distributions across the tubule [30]. Since rat *apela* is not annotated in the rat reference DNA databases in the original mapping of RNA-Seq reads we looked specifically in the mouse *apela* sequence to find the corresponding rat homologue; the distribution of *aplnr* and *apelin* in the same kidney structures was similarly extracted. Details of the methods and analysis have been outlined [30].

### Statistical analysis

IN Cell Analyser 1000 experiments were performed in triplicate wells, with experiments performed at least 2 times. Statistical analysis was with a one-way ANOVA and *post hoc* Dunnett’s test with GraphPad Prism software (version 4.0b). *p*<0.05 was considered as statistically significant.

## Results

### Studies on CHO cells

**Fig 1:**
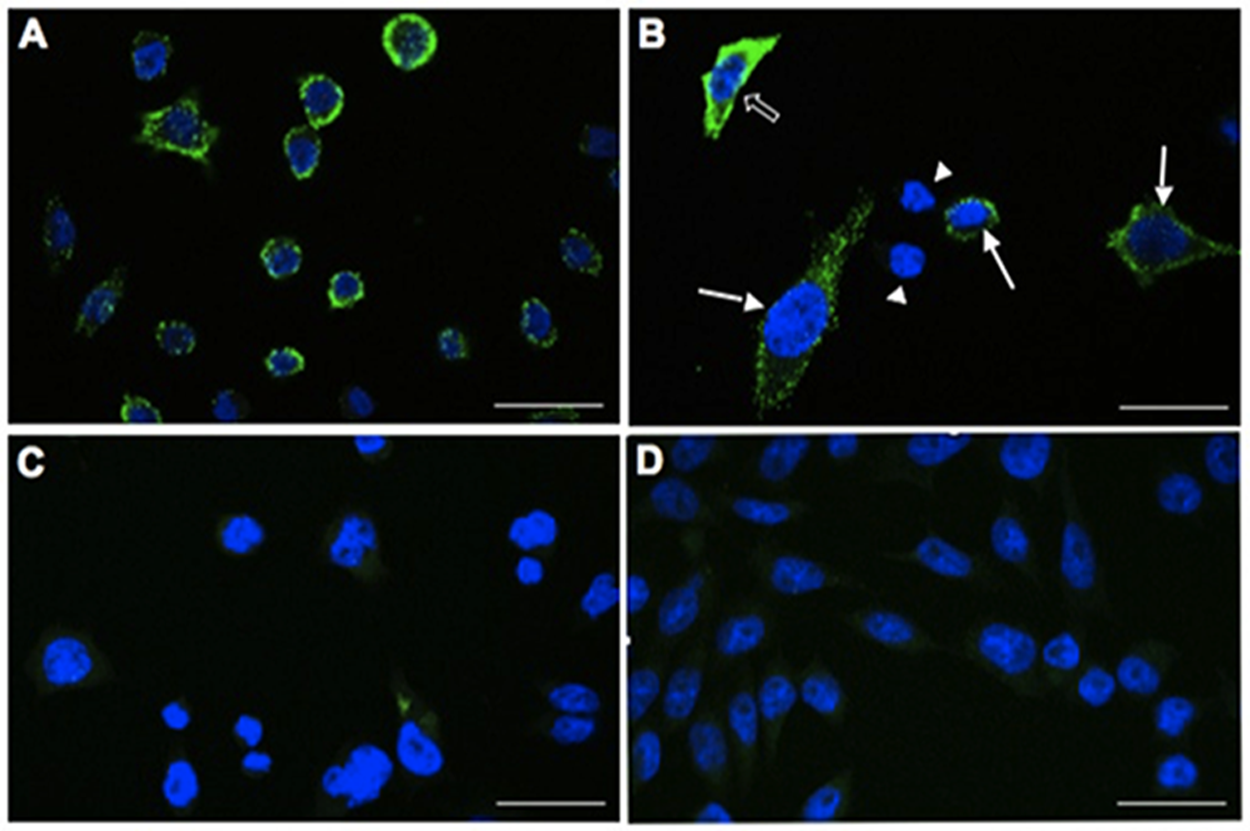
RNAscope with *aplnr* probes in CHO-B78 (rat APJ) and non-transfected CHO cell cultures. In **(A)** clear, punctate *aplnr* labelling is present in the cytoplasm of the majority of CHO-B78 cells with cell-cell variability in the levels of expression. Strongly (open arrow), moderately (arrows) and unlabelled (arrowheads) *aplnr* cells in CHO-B78 cultures are shown in (**B**). **(C)** shows negative control probe on CHO-B78 cells, while the lack of *aplnr* labelling in non-transfected CHO cells is shown in **(D)**. Scale bar = 25μm.

Studies on CHO cells stably transfected with the rat *aplnr* cDNA (B78) were used as a positive control for the *aplnr* probe, confirming expression of APJ, and functional activity of apela-32 at APJ, in this cell line. *Aplnr* mRNA is highly expressed as punctate dots in CHO-B78 cells (Fig 1A). The dots merge in areas of high *aplnr* expression (e.g., see Fig 1B). There is some variability in cell-cell *aplnr* expression and occasional cells do not appear to exhibit any *aplnr* expression (<5% from a count of 81 cells; see Fig 1B). No signal is detected in CHO-B78 cells hybridized with negative control probes (Fig 1C)—similarly *aplnr* expression is not observed in non-transfected CHO cells (Fig 1D).

**Fig 2:**
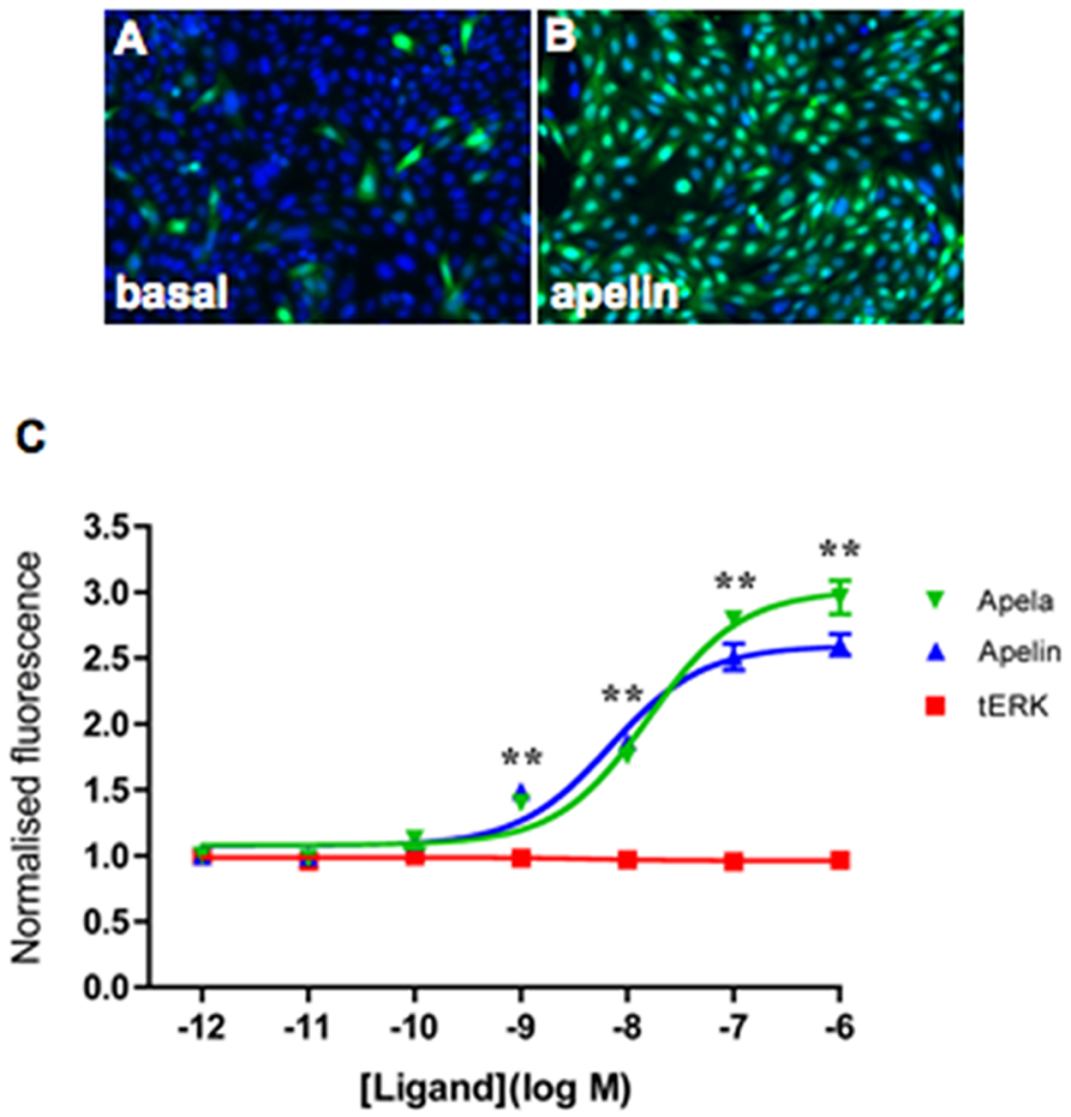
[Pyr^1^]apelin-13 and apela-32 activates ERK1/2 in CHO-B78 cells. Cells were treated for 10min at 37°C with the indicated concentration of ligands. Representative thumbnail images acquired by the IN Cell Analyzer 1000 show immunohistochemical staining for intracellular ppERK1/2 (green) after treatment of CHO-B78 cells with vehicle (**A**) or 100nM [Pyr^1^]apelin-13 (**B**). The workstation can automatically demarcate nucleus from cytoplasm according to DAPI (blue) nuclear staining. A dose-response curve (whole-cell immunofluorescence expressed in arbitrary units) for [Pyr^1^]apelin-13 (blue) or apela-32 (green) is shown in (**C**). There are no measurable dose-dependent changes in tERK levels (red) in cells stimulated with apela-32—a similar result was obtained with cells stimulated with [Pyr^1^]apelin-13. The [Pyr^1^]apelin-13 and apela-32 stimulations were performed in separate cell wells (cells were used from the identical CHO-B78 passage number) and processed for ERK immunohistochemistry in the same experiment. Data are expressed as mean ± SEM, averaged from two separate experiments, each ligand concentration or vehicle (basal) with triplicate wells, and at least triplicate fields within wells. **p<0.01 comparing stimulations to basal conditions.

Apela-32 and [Pyr^1^]apelin-13 stimulate ERK activation (*p*<0.01) in CHO-B78 cells in a dose-responsive manner between 10^−10^–10^-6^M (Fig 2). The EC50’s are 6.9nM and 15.9nM for [Pyr^1^]apelin-13 and apela-32, respectively.

### Apela distribution in rat and mouse kidney

#### ISHH with radiolabelled oligonucleotides

**Fig 3:**
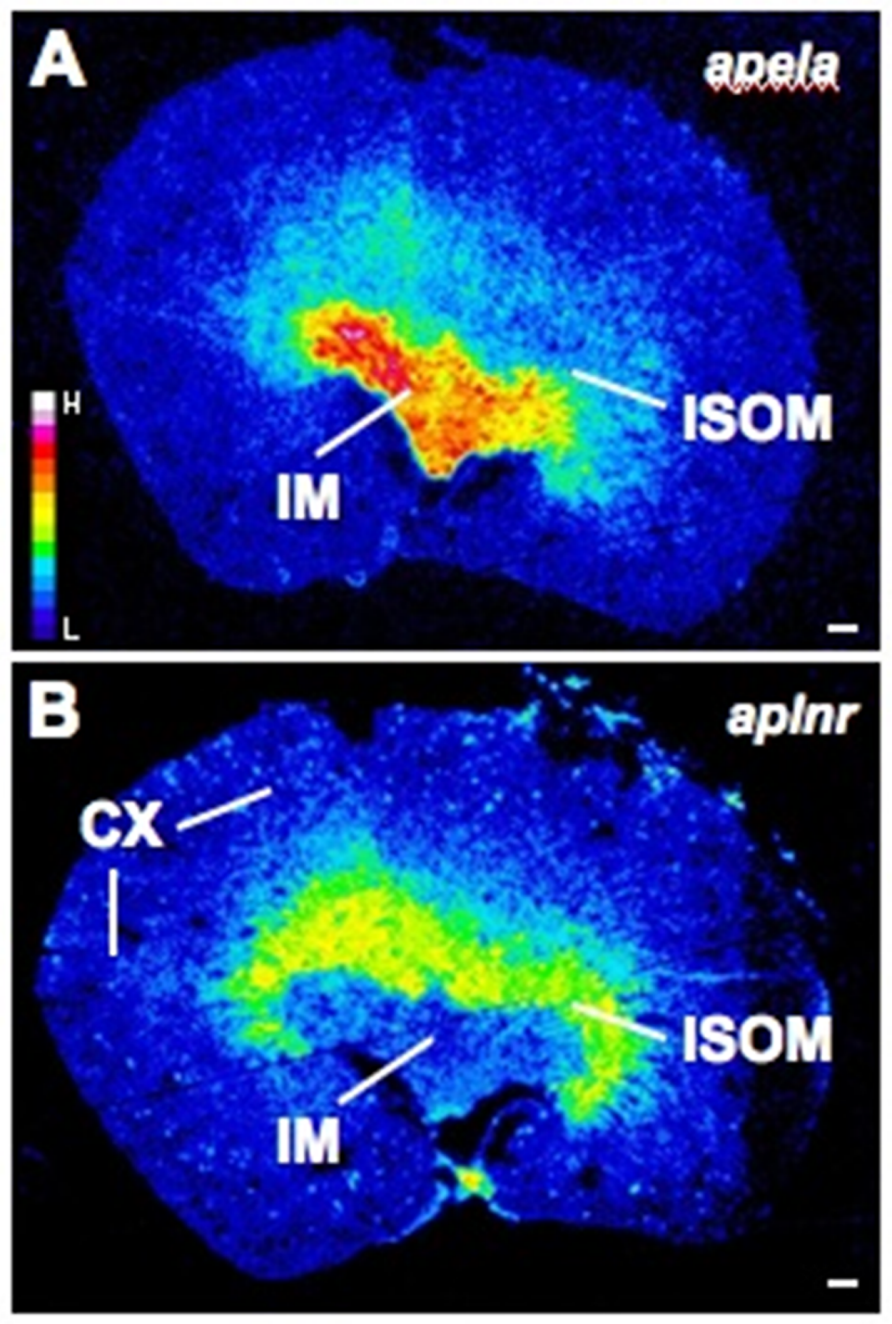
Representative autoradiographic film images demonstrating *apela* (A) and *aplnr* (B) expression in serial sections of a kidney from an adult male Wistar rat. *Apela* is highly expressed in the inner medulla (IM) whereas *aplnr* labelling is primarily in the inner stripe of the outer medulla (ISOM). In contrast to the *apela* labelling, the *aplnr* probes also label a subpopulation of glomeruli (patchy ‘dots’) in the cortex (CX). The film images were exposed to film for 3 months, developed, scanned, exported to Image J and pseudocoloured. In these images yellow-red designates high expression whereas blue-black represents negligible or no labelling (see pseudocolour scale bar in (**A**)). The images are representative of results obtained from the kidneys of 8 rats. Scale bar = 500μm.

The major site of *apela* expression in the rat kidney is the inner medulla (IM) with weaker expression in the inner stripe of the outer medulla (ISOM) (Fig 3A and 3B). Serial sections of the same kidneys hybridized with *aplnr* probes show a pattern in the medulla that is inverted compared to *apela* expression—*aplnr* is more highly expressed in the ISOM than IM. *Aplnr* is also expressed in glomeruli, visualized as a patchy, ‘speckled’ pattern in the cortex.

**Fig 4:**
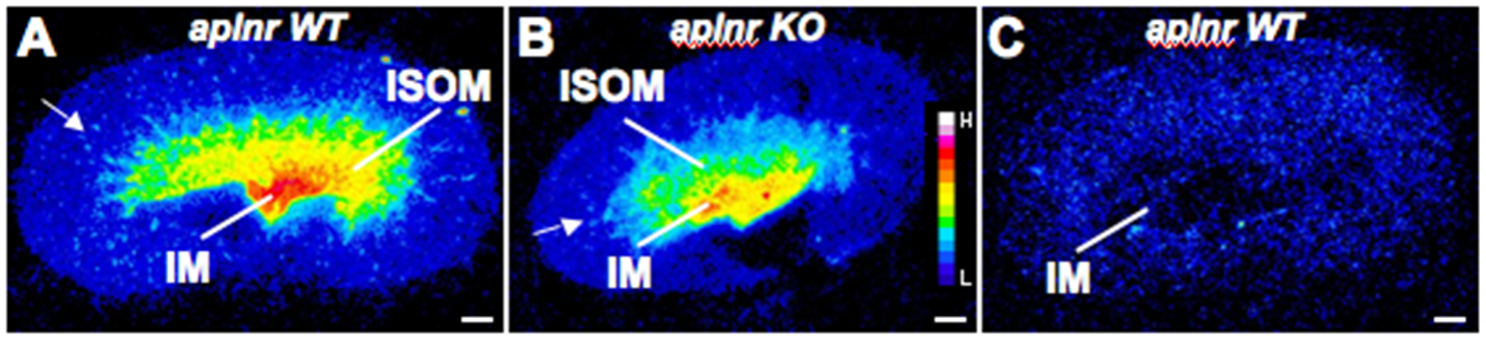
Autoradiographic film images of ISHH with antisense-*apela* probes in kidneys from adult male *aplnr* wildtype (WT) (A,C) and knockout (KO)(B) mice. As in the rat kidney, *apela* labelling in the mouse kidney is mainly located in the inner medulla (IM) with weaker labelling in the inner stripe of the outer medulla (ISOM). There is also weak, scattered labelling in the cortex (arrowed). Background sense *apela* probe labelling is shown in (**C**), where the image contrast was increased in Image J to show the outline of the tissue. Pseudocolour scale bar is shown in (**B**). The sections were exposed to film for 5 weeks and images were processed as in Fig 3. Scale bar = 500μm.

In the mouse kidney (Fig 4A and 4B) the *apela* pattern of labelling is similar to that observed in the rat. In the cortex of both mouse and rat kidneys there was some light labelling of isolated structures. There is no apparent difference in the distribution of *apela* between *aplnr* wildtype and knockout mice (Fig 4A and 4B). Sense *apela* probes give uniform background labelling in the kidney (Fig 4C).

**Fig 5:**
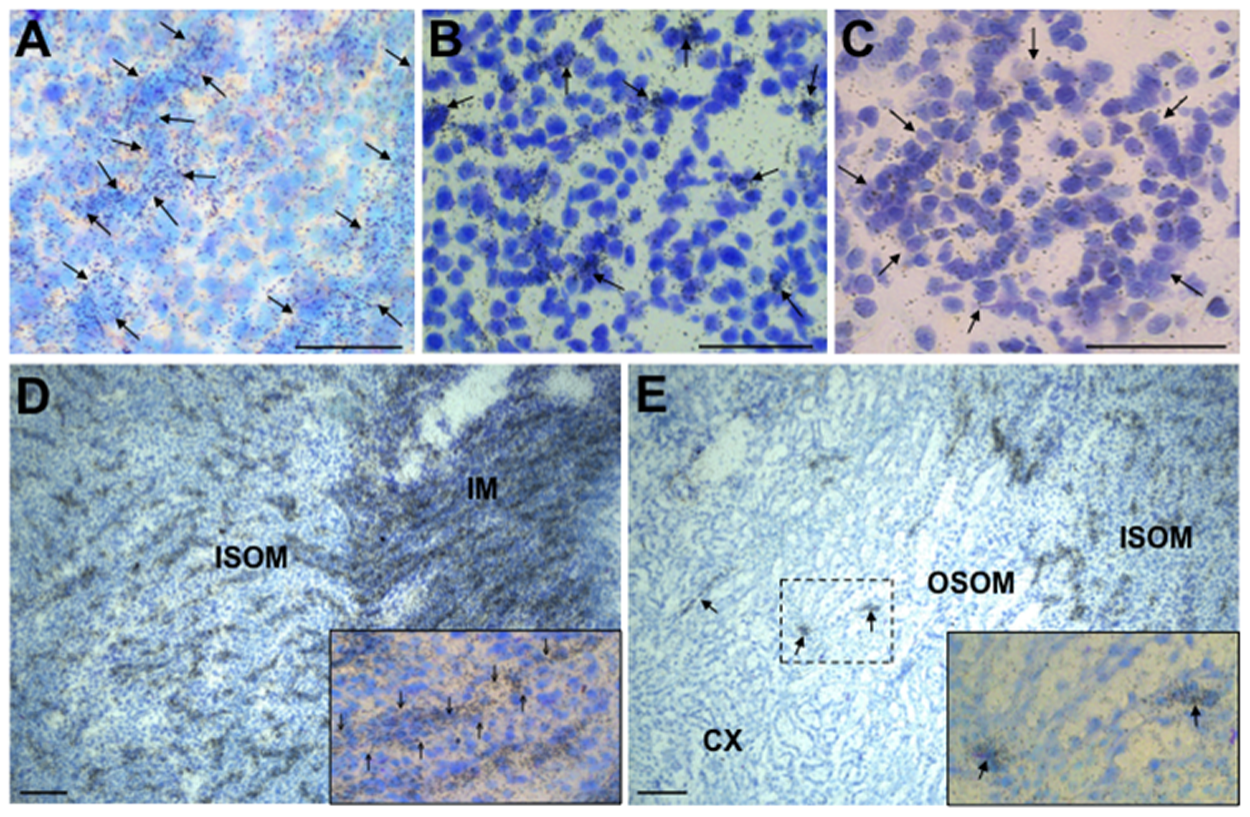
Brightfield images of ISHH labelling of *apela* and *aplnr* transcripts (black grains) in adult rat and mouse kidney. *Apela* is largely confined to tubular structures (outline of some arrowed in (**A**)) whereas *aplnr* is mainly expressed in intertubular cells (arrowed in (**B**)) in the rat inner medulla (IM) and outer medulla, respectively. Approx. 35% of cortical glomeruli also expressed *aplnr*, usually in a uniform distribution across the structure (perimeter of glomerulus arrowed)—an *aplnr*-positive rat glomerulus is shown in (**C**)). There is a clear demarcation in overall labelling in tubular structures in the transition from inner medulla (IM) to inner zone of the outer medulla (ISOM) (**D**) and from the outer medulla (tubular structures arrowed in the outer stripe of the outer medulla (OSOM)) to the cortex (CX) (**E**) in mouse kidneys. In **D** and **E** the insets are approximately 4-fold magnifications of the wide-field images, showing *apela* labelling in tubules (one structure arrowed) in the ISOM (**D**) and isolated cells (from boxed area) in the OSOM (**E**). Scale bar = 50μm in **A**-**C**, and 100μm in **D** and **E**.

In emulsion-dipped sections of rat and mouse kidney *apela* labelling was pronounced in tubules, particularly in the inner medulla collecting ducts (Fig 5A) whereas *aplnr* was most prominently expressed in scattered, intertubular (interstitial) cells in the medulla (Fig 5B) and in glomeruli in the cortex (Fig 5C). There was a stark transition, from high to low, in the number of labelled tubular structures between the IM and outer medulla (Fig 5D), and outer medulla and cortex (Fig 5E).

#### Triple labelling of *apela*, *apelin* and *aplnr* cells using branched-chain ISHH

**Fig 6:**
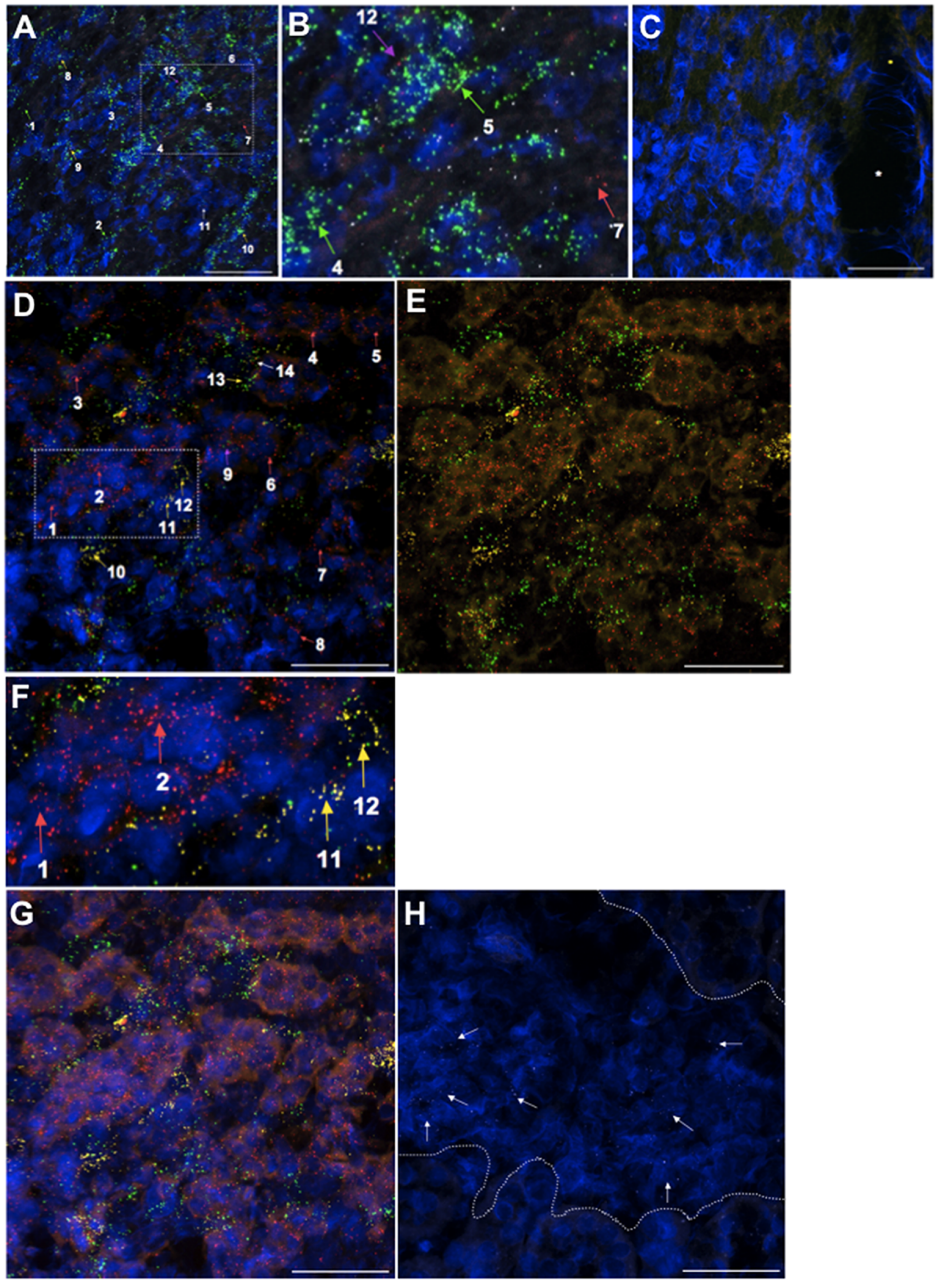
*Apela*, *apelin* and *aplnr* expression in the adult rat heart ventricle and kidney by branched-chain ISHH. The tissues were hybridized with RNAscope probes and processed in one day. Positive *apela*, *apelin* and *aplnr* and negative control staining of the heart is shown in (**A, B**) and (**C**), respectively. In (**A**) *aplnr*-expressing cells (green dots; labelled 1–6 with green arrows) are widely distributed in the heart myocardium. The rare cells express only *apela* (red dots; labelled 7 with a red arrow). Some cells are labelled with both *aplnr* + *apelin* (white dots) probes (labelled 8–10 with yellow arrows). A few cells express *aplnr* + *apela* + *apelin* (labelled 11 with a white arrow) or *aplnr* + *apela* (labelled 12 with a purple arrow). (**B**) is an enlargement of the dotted area outlined in (**A**). There was no labelling with negative control probes in all three channels in the heart ventricles (**C**) where * denotes vessel. In the kidney inner medulla (**D**) the vast majority of cell expressing *apela* (red dots; labelled 1–8 with red arrows) do not express *aplnr* (green dots) or *apelin* (yellow dots). Some cells express both *aplnr* + *apela* transcripts (labelled 9 with a purple arrow) while *aplnr* + *apelin* cells are more common (labelled 10–13 with yellow arrows). The rare cell exhibits *apela* + *apelin* + *aplnr* labelling (14; white arrow). Removing the DAPI channel in (**E**) (corresponding image of (**D**)) highlights the distinction between *apela* (red) and *aplnr*/*apelin* expression (green and yellow, respectively) in the kidney. (**F**) is an enlargement of the dotted area outlined in (**D**). In (**G**) the red channel diffuse background is enhanced to clearly show the renal tubular *apela* (red) labelling. (**H**) shows *aplnr* expression (white dots; e.g., arrowed) in a glomerulus (partial boundaries shown by dotted line). The images are representative of results obtained from the hearts and kidneys of 3 and 4 rats, respectively. DAPI counterstaining is shown in blue. Scale bar = 50μm.

All three genes are expressed in the adult rat heart (Fig 6A), used as positive tissue control for *aplnr* and *apela* detection. Some dots are pinpoint while others are larger and appear to be a combination of more than one dot. *Aplnr* is highly expressed in cardiomyocytes throughout the myocardium in a pattern largely distinct from that of the more sparsely distributed *apelin* cells and the even less abundant *apela* cells (Fig 6A and 6B). The majority of cells appear to express *aplnr* alone, however in some cells *aplnr* and *apelin* appear to be colocalized. The occasional cells express all three genes, *aplnr* and *apela*, or *apelin* or *apela* alone. Most *apela*-positive cells contain only a few dots, in contrast to up to 20 dots observed in *aplnr*-positive cells. Negative control probes do not label (Fig 6C).

The relative number of *apela*-, *apelin*- and *aplnr*-expressing cells in the heart substantially differs from that observed in the adult rat kidney, where there are many more cells expressing *apela*, particularly in the IM and ISOM, than *aplnr* or *apelin* (Fig 6D). In agreement with our ISHH results using radiolabelled probes, rat kidney *apela* expression is largely confined to tubular elements (Fig 6D–6G), and is particularly pronounced in the IM. The vast majority of *aplnr* and *apelin* expression in cells do not overlie *apela*-expressing cells (Fig 6D–6G). While most *aplnr*-positive cells also appear to express *apelin*, a few cells express *aplnr* or *apelin* alone. As in the heart many cells express only single digit numbers of *apela*, *apelin* or *aplnr* dots.

We did not observe any *apela* or *apelin* labelling of glomeruli, while about 35% (17 out of 49 viewed from kidneys of 4 separate rats) of glomeruli express *aplnr* (only 1–5 dots/cell) in a diffuse pattern in glomerular cells (Fig 6H).

#### Mining of transcriptome (RNA-Seq) data of microdissected rat renal tubules and glomeruli

*Apela*, *apelin* and *aplnr* show distinct patterns of expression along the tubule (Table 1). Notably *apela* is expressed throughout the tubule with strongest expression in the Loops of Henle and inner medullary collecting duct (IMCD), whereas *apelin* is absent from the tubules. Low levels of *aplnr* are present in the distal convoluted tubule (DCT) and cortical thick ascending loop of Henle (cTAL)—*aplnr* is not detected in collecting ducts. The RNA-Seq data does not include extra-tubular elements such as vasa recta where ISHH shows *aplnr* to be highly expressed. It confirms and extends the tubular expression of *apela* obtained by ISHH.

**Table 1:** Relative mRNA abundance for *apela*, *apelin* and *aplnr* quantified by RNA-Seq in microdissected rat kidney tubules.

	P S1	P S2	P S3	SDL	LD LOM	LD LIM	tAL	mTAL	cTAL	DCT	CNT	CCD	OMCD	IMCD
*Apelin*	0	0	0	0	0	0	0	0	0	0	0	0	0	0
*Aplnr* *(APJ)*	0	0	0	0	0	0	0	0	0.1	0.2	0	0	0	0
*Apela*	0	0	0.5	15.3	17.6	34.1	15.9	0.4	0.5	1.2	1.1	3.7	5.5	17.5

Gene expression levels listed are maximum reads across the gene body [30]. The RefSeq IDs are NM_031612 (*apelin*) and NM_031349 (*aplnr)*. Full data is at https://hpcwebapps.cit.nih.gov/ESBL/Database/NephronRNAseq/ but does not include *apela* because it was unannotated (with no RefSeq ID) in rat. Tubule analysis excludes interstitial (e.g., interstitial cells, blood cells) and vascular elements (e.g., endothelial cells) where there is *Aplnr (APJ)* expression (e.g., in the ISOM). It appears that *apela* markedly outstrips *apelin* in terms of expression level. *Apela* appears to be expressed in all renal tubule segments beyond the proximal tubule and is more highly expressed in medullary segments than in cortical segments. Abbreviations: Proximal tubule (P): S1; directly attached to glomeruli; S2; straight part in medullary ray; S3; in outer medulla; SDL, Short descending limb of the loop of Henle; LDLOM, Long descending limb of the loop of Henle (in outer medulla); LDLIM, Long descending limb of the loop of Henle (in inner medulla); tAL, Thin ascending limb of the loop of Henle; mTAL, Medullary thick ascending limb of the loop of Henle; cTAL, Cortical thick ascending limb of the loop of Henle; DCT, Distal convoluted tubule; CNT, Connecting tubule; CCD, Cortical collecting duct; OMCD, Outer medullary collecting duct; IMCD, Inner medullary collecting duct.

## Discussion

APJ was an orphan GPCR [23, 31] until its endogenous ligand apelin was isolated from stomach extracts by ‘reverse pharmacology’ almost 20 years ago [32]. It has recently been proposed that apela, a distinct protein unrelated to apelin, and highly conserved across vertebrates [8,9], is a new endogenous ligand for APJ. We have confirmed previous studies showing that apela stimulates ERK activation in APJ-expressing cells *in vitro* [11]. Using ISHH to highlight gene expression in an anatomical context we show that *apela* mRNA is not abundantly expressed in the adult rat heart, and that there are relatively more *apela*-expressing cells in the inner, compared to the outer, medulla of the rat and mouse kidney, whereas the converse is true for *aplnr* in rats. In addition, we show *apela* to be mainly found in tubular elements in contrast to *aplnr* (and *apelin*); findings that are supported by RNA-Seq data on microdissected rat tubular segments. In the renal medulla, *apelin* expression is aligned with *aplnr* expression, where some cells appear to express both genes.

In the present study the cardiac expression of *apela* is particularly low, with only sparse cells expressing 1–3 mRNA transcripts/cell, which is in contrast to reports of a more widespread *apela* expression as demonstrated by RT-PCR in endothelial cells, fibroblasts and cardiomyocytes isolated from adult rat heart [20]. However this result is consistent with recent studies showing apela-immunoreactivity predominantly in vascular endothelium in the ventricles of human heart [33]. In zebrafish, *apela* is expressed primarily in the embryonic heart [10] and appears to be the main APJ ligand in promoting cardiac development [8,9]. *In vivo* both apelin and apela increase cardiac contractility in adult rodents and humans [2,20,33,34,35]. Identification of apela protein expression in rat and mouse tissues may be facilitated by the availability of apela-specific antibodies that are suitable for immunohistochemistry in these species. In addition, global or apela-focused proteomic approaches concentrating on short peptides (such as apela) may also be used to identify precise apela proteoforms. These proteins may exhibit biased agonism/functional selectivity as has been demonstrated for the various apelin proteoforms *in vitro* [34,36]. Apela, like apelin, may also signal to ERK or other signalling molecules via G protein-dependent and -independent pathways [35,37]. It is possible that *apela* RNA acts *in vivo* as an APJ-independent regulatory ncRNA, although this has been shown to be functionally relevant in only one system (apoptosis in mouse ESCs [19]) to date.

The expression of genes for one or both APJ-ligands with their receptor in the same cell, or cells expressing either ligand gene in the close proximity of APJ-expressing cells, raises the possibility of autocrine and/or paracrine actions for the apelinergic system within the heart and kidney. The demonstration of co-localization of receptor/ligand pairs in individual cells is not without precedent, e.g., in the brain a GPCR may regulate the release of its own endogenous ligand co-expressed in the same (or neighbouring) cell [38]. *Aplnr* and *apelin* appear to be co-localized in endothelial cells in and around microvascular proliferations in brain tumour specimens [39] and we speculate that most *aplnr* and *apelin* co-expression in the kidney is likely to be in vascular elements. Similarly, while *aplnr*-*apelin*-*apela* co-expression is rare in the kidney, the co-localization of multiple receptor-ligand combinations (e.g., for the chemokine receptor/ligand family [40]) in other tissues such as brain has been described, and is thought to contribute to plasticity in responses to diverse physiological stimuli. Although apela has high affinity for APJ and it may be assumed that when exogenously administered it binds to all APJs, it may not always be active at APJ. For example, whereas *aplnr* (and *apelin*) knockdown alters the expression of the GPCR *CXCR4* in cultured human umbilical vein endothelial cells, apela stimulation or knockdown has no effect [41]. In the kidney it would appear that many of apela’s effects (e.g., on diuresis [11]) are mediated by APJ present in glomeruli and/or outer medullary vascular elements. Intravenous apelin-17 also causes diuresis and effects haemodynamic function in microdissected glomeruli *in vitro* [22]. The relative abundance of *apela* over *apelin* in the kidney may suggest that renal apela (assuming that it is translated into functional protein) is the more important APJ ligand in regulating renal function—this of course does not take into account possible effects of circulating levels of apela and/or apelin. As measured by enzyme immunoassay, apela has been detected in human plasma at slightly higher levels than apelin [33]. We note that low levels of *aplnr* expression have been reported by others in microdissected collecting ducts [22], an obvious site for exogenous or circulating apelin/apela’s diuretic effects, whereas we do not observe significant tubular expression of *aplnr*. We propose that renal apela in the collecting duct and other tubular elements may access extra-tubular APJ in structures like the vasa recta to regulate the osmotic gradient and blood flow throughout this region. There is close proximity of tubular and vascular elements in the medulla, and cross-talk involving these structures has been implicated in the function of other peptide-receptor systems involving vasoactive agents, including endogenous peptides such as endothelin-1 [42,43] that is expressed throughout the nephron, collecting ducts and renal vasculature with its cognate GPCRs ETA and ETB [44]. We cannot exclude the possibility that renal apela disseminates into the blood to function as an endocrine hormone (e.g., like erythropoietin) and/or acts locally or at extra-renal sites on another, currently unidentified receptor, as proposed for its anti-apoptotic action in human ESCs [18].

Many questions remain to be explored. For example, does apela in its ncRNA or peptide form(s) contribute to the pathological dispositions of some cardiovascular and metabolic conditions? Apelin and apela have anti-apoptotic activity in a number of tissues and cells [2,19] and both apelin and apela have been implicated in cardiac pathophysiology and cardiovascular disease [1,2,34]. For example, vascular endothelium *apela* mRNA and/or apela levels are reduced in pulmonary arterial hypertension in human patients and rat models, and apela-32 attenuates the remodelling of pulmonary vasculature and hypertrophy in right ventricular cardiomyocytes [33]. In addition apela may participate with apelin in renal protection against fibrosis, ischaemia and diabetic nephropathy [45]. Studies on how *apela* expression is regulated (in concert with *apelin* and/or *aplnr* expression where the genes co-exist) may also be informative as to which APJ ligand may be active in target tissues. From a functional viewpoint, based on studies in zebrafish [8,9], the majority of *apela* knockouts in rodents may not be viable and so genetic manipulation of *apela* will likely require the use of inducible, conditional knockouts or RNA-edited models, and/or RNA interference with *apela*-specific knockdown constructs, to unravel its relative importance compared to apelin in regulating renal function.

Despite the fact that *apela* mRNA transcripts have been reported to be detected ‘exclusively’ in adult rat kidney [11], our study highlights the utility and sensitivity of branched-chain ISHH to rapidly detect *apela* mRNA expression in isolated cells in other tissues such as the heart. The full spectrum of *apela*-expressing tissues and the phenotype of the cells therein, and the functional relevance of apela as a separate entity or in unison with apelin, awaits determination. These and other questions will benefit from a thorough characterization of the sites of *apela* expression in a cellular context.
